# Editorial: Current research and emerging directions on the cognitive and neural organization of speech processing

**DOI:** 10.3389/fnhum.2015.00305

**Published:** 2015-05-27

**Authors:** Patti Adank, Carolyn McGettigan, Sonja A. E. Kotz

**Affiliations:** ^1^Division of Psychology and Language Sciences, Speech, Hearing and Phonetic Sciences, University College LondonLondon, UK; ^2^Department of Psychology, Royal Holloway University of LondonEgham, UK; ^3^Max Planck Institute LeipzigLeipzig, Germany; ^4^School of Psychological Sciences, University of ManchesterManchester, UK

**Keywords:** speech perception, speech production, functional magnetic resonance imaging (fMRI), magnetoencephalography (MEG), electroencephalography, transcranial magnetic stimulation (TMS)

This Research Topic consists of 14 manuscripts discussing the cognitive and neural organization of speech processing. The contributions are grouped around four themes: (1) Spoken language comprehension under difficult listening conditions; (2) Sub-lexical processing; (3) Sensorimotor processing of speech; (4) Speech production.

Seven papers addressed speech perception under challenging listening conditions. Van Engen and Peelle (2014) discuss the effects of processing speech in an unfamiliar regional or foreign accent. They argue that, as perceiving accented speech incurs a processing cost, just like other types of distortions such as background noise, it should also be regarded as representing a challenging listening condition. Neger et al. (2014) focused on plasticity of speech processing in statistical and perceptual learning tasks in aging. They conclude that perceptual and statistical learning share mechanisms of implicit regularity detection, but that the ability to detect statistical regularities is impaired in older adults for fast visual sequences. Dekerle et al. (2014) examined whether speech perception in a multi-speaker background relies on semantic interference between the background and target speaker using a semantic priming paradigm in three experiments. Their results indicate that higher-level linguistic processes such as semantic priming may not be as automatic as commonly thought but are subjected to the limits of cognitive resources such as working memory and attention. Yi et al. (2014) evaluate how processing of foreign-accented speech relates to social cognition. It was concluded that foreign-accented speech perception engages greater activation of neural systems underlying speech perception, and that implicit Asian-foreign association is related to with decreased neural efficiency in early spectrotemporal processing. Vitello et al. (2014) used fMRI to address the question of how semantic ambiguities are resolved during speech comprehension.

Strauß et al. (2014) examined through literature review whether neural oscillations in the alpha frequency range (~ 10 Hz) act as a neural mechanism to selectively inhibit the processing of noise to improve auditory selective attention to task-relevant speech signals. Ding and Simon (2014) discuss whether cortical entrained activity is related more closely to speech perception or to auditory encoding that is not specific to speech, by reviewing evidence regarding various hypotheses about the functional roles of cortical entrainment to speech.

Three papers focused on perception of speech at sub-lexical levels. Deschamps and Tremblay (2014) studied perception of sub-lexical information by examining the neural bases of processing of simple syllables and more complex syllabic structures using fMRI, while Yu et al. (2014) used MEG to study the neural processing of disgust in anterior insula by presenting listeners with syllables with differed intended emotional meanings. Finally Chen et al. (2014) investigated processing of acoustic and phonological information in lexical tones in Mandarin Chinese using EEG.

Two papers addressed sensorimotor processing of speech. Komeilipoor et al. (2014) report higher motor excitability as measured using Transcranial Magnetic Stimulation (TMS) in the tongue area during the presentation of meaningful gestures (noun-associated). Sowman et al. (2014) demonstrate that appropriately timed TMS to the hand area, paired with auditorily mediated excitation of the motor cortex, induces an enhancement of motor cortex excitability that lasts beyond the time of stimulation.

Two papers focused on speech production. Etchell et al. (2014) provide a review of the stuttering literature and Hernandez-Pavon et al. (2014) present a neuronavigated TMS study exploring the neural locus of aspects of picture naming in healthy participants.

This Frontiers Research Topic allows new insights into the neurobiology of speech perception and production, and demonstrates how the field of speech science is now addressing issues at its very core. We believe that the future of the research in the field lies in the effective combination of research methods, e.g., EEG and TMS, or fMRI and EEG, as research will benefit from the strengths of each method. In conclusion, this Research Topic consists of 14 excellent contributions, and we are convinced the Topic will provide readers with novel ideas for future studies that will elucidate the cognitive and neural architecture of speech processing.

## Conflict of interest statement

The authors declare that the research was conducted in the absence of any commercial or financial relationships that could be construed as a potential conflict of interest.
